# Negative beliefs about low back pain are associated with high pain intensity and high level disability in community-based women

**DOI:** 10.1186/1471-2474-9-148

**Published:** 2008-11-04

**Authors:** Donna M Urquhart, Robin J Bell, Flavia M Cicuttini, Jisheng Cui, Andrew Forbes, Susan R Davis

**Affiliations:** 1 Department of Epidemiology and Preventive Medicine, Monash University, Alfred Hospital, Melbourne, Australia; 2 Women's Health Program, Department of Medicine, Monash University, Alfred Hospital, Melbourne, Australia; 3 World Health Organization Collaborating Centre for Obesity Prevention, Deakin University, Melbourne, Australia

## Abstract

**Background:**

Although previous studies have investigated beliefs about back pain in clinical and employed populations, there is a paucity of data examining the beliefs of the broader community. We aimed to characterize the beliefs that community-dwelling women have about back pain and its consequences, and to determine whether those with varying levels of pain intensity and disability differ in their beliefs.

**Methods:**

542 community-dwelling women, aged 24 to 80 years, were recruited from a research database. Participants completed a self-administered questionnaire that included detailed demographic information, the Chronic Pain Grade Questionnaire (CPG) and the Back Beliefs Questionnaire (BBQ). The CPG examined individuals' levels of pain intensity and disability, and the BBQ investigated their beliefs about back pain and its consequences.

**Results:**

506 (93.4%) women returned the study questionnaire. The mean (SD) BBQ score for the cohort was 30.7 (6.0), indicating generally positive beliefs about back pain. However, those women with high intensity pain and high level disability had a mean (SD) score of 28.5 (5.7) and 24.8 (5.7) respectively, which reflects greater negativity about back pain and its consequences. There was an association between negative beliefs and high pain intensity (OR = 0.94 (95% CI: 0.90, 0.99), p = 0.01) and high level disability (OR = 0.93 (95% CI: 0.89, 0.97), p = 0.001), after adjusting for confounders.

**Conclusion:**

This study highlights that although women living in the community were generally positive about back pain, subgroups of women with high pain intensity and high level disability were identified who had more pessimistic views. While a causal relationship cannot be inferred from these cross-sectional data, the results suggest that negative beliefs individuals have about back pain may be predictive of chronic, disabling spinal pain.

## Background

Chronic low back pain is an economic and social burden world-wide [1]. While most people will experience low back pain during their lifetime [2], much of the suffering and financial costs are associated with the 5–10% of people that experience associated disability [3]. Hence, the need to identify factors associated with disabling low back pain so that targeted prevention strategies for primary health care can be developed.

Biological, psychological and social factors have been recognised as having a significant impact on the persistence of pain and the development of disability [4]. In particular, an individual's belief system about pain is considered to be important [5]. Irrational beliefs about back pain, in which the pain is considered to be a signal of impending threat, can result in fear of movement/(re)injury, reduced function and activity, and subsequently maintenance and exacerbation of chronic disability [5].

There is evidence that educational strategies are effective in changing beliefs about back pain and reducing fear and associated disability. Clinical trials have reported reductions in both persistent low back pain [6] and disability [7] in primary care patients following implementation of educational booklets designed to change misconceptions about back pain. Moreover, a public health prevention program implemented in an employed population in the Australian state of Victoria not only resulted in patients and doctors developing more positive beliefs about back pain, but a decline in the number of claims, rates of days compensated and medical payments for back pain was reported [8].

Although these studies have investigated beliefs about back pain in clinical and employed populations, there is a paucity of data pertaining to the beliefs of individuals living in the broader community. This is the case even though a Canadian study, which surveyed 2,400 individuals from two provinces, reported that generally pessimistic beliefs about low back pain were held by the population [9]. Moreover, there are limited studies examining the relationship between beliefs and both low back pain and disability in community-based cohorts. Such information is important if are to understand the predictive nature of beliefs in the general population and subsequently design and tailor education strategies to communities' needs. The aims of this study were to characterize the beliefs of community-dwelling women about back pain and its consequences, and determine whether individuals with varying levels of pain intensity and disability differ in their beliefs about back pain.

## Methods

### Study sample

We recruited participants from a community-based research database that was established through random sampling from the Victorian Electoral Roll between April 2002 and August 2003. Detailed information about recruitment of women from this database to a large cross-sectional study of androgens in women has been previously reported [10]. Participants in this study of androgens who agreed to be contacted regarding further research were invited to participate in the current study. No incentives were offered to the subjects. Of the 754 women who were re-contacted, 542 (71.9%) agreed to be involved.

### Questionnaire

#### Demographic and prevalence data

Data were obtained for age, weight and height, smoking, alcohol consumption, and menopause, relationship and employment status. Body mass index (BMI; kg/m^2^) was calculated from height and weight measurements provided by the participants. Women were classified as 'partnered' if they were married, in a de-facto relationship or single with a partner. Employment was defined as work outside the home and included full-time, part-time, casual and shift work. Women were categorized into 3 groups, premenopausal, perimenopausal and postmenopausal, using a hierarchy of information including history of a bilateral oophorectomy, age (≥ 60 years), current use of hormonal contraception or systemic hormone therapy, history of a hysterectomy, bleeding pattern and the presence of vasomotor symptoms [10]. Outliers that were identified in the demographic data were cross-checked with data from the previous cross-sectional study of androgens to ensure they represented meaningful values.

#### Low back pain prevalence

To obtain data on low back pain prevalence, participants were asked if they had experienced low back pain at the following time points/periods; now, past 6 months, past 12 months or ever. A body chart was provided, with the region from the border of the rib cage to the gluteal folds shaded, to define the region of pain.

#### Pain intensity and disability data

To obtain data on low back pain intensity and disability the Chronic Pain Grade Questionnaire (CPG) was administered. The CPG has been shown to be a reliable and valid instrument for use in population surveys of chronic pain, including low back pain [11,12]. The CPG includes 7 questions from which a pain intensity score (0–100) and disability score (0–6) are calculated.

Based on these scores, participants are classified into one of the CPG grades:

Grade 0 – no pain or disability

Grade 1 – low pain intensity (and low disability)

Grade 2 – high pain intensity (and low disability)

Grade 3 – high disability, moderately limiting (regardless of pain intensity)

Grade 4 – high disability, severely limiting (regardless of pain intensity)

To investigate the relationship between pain intensity and beliefs about back pain, participants were classified into 3 groups based on their pain intensity score; no pain (= 0), low pain intensity (<50) and high pain intensity (≥ 50). Subjects were also categorized into 3 groups based on their disability score; no disability (= 0), low disability (<3) and high disability (≥ 3) and these groups were used to examine the relationship between disability and beliefs.

#### Beliefs about back pain

The Back Beliefs Questionnaire (BBQ) was used to examine individual's beliefs about back pain and its consequences, regardless of whether back pain had been previously experienced. The questionnaire has been reported to have good internal consistency (Cronbach: 0.7) and test-retest reliability (ICC: 0.87) [13]. The questionnaire consists of 14 statements to which the respondent indicates their level of agreement on a 5 point scale. A score of 1 indicates complete disagreement and a score of 5 complete agreement. As 5 of the 14 statements are distractors, the scores of the 9 remaining statements are reversed and then summed to provide a total score ranging from 9 to 45. A lower score indicates the respondent has more negative beliefs about back pain. Two population-based studies undertaken in Australia [8] and Canada [9] have previously used the BBQ, and prior to the implementation of a public health campaign, reported mean back belief scores of 26.5 (95% CI: 26.1, 26.8) and 26.4 (SD: 6.4) respectively.

### Procedures

A study package including the participant information sheet, consent form and study questionnaire was mailed to participants in July 2006. Participants that did not return their questionnaire were sent a letter to ask whether they have received the questionnaire and if they needed assistance with its completion. If participants did not respond to this letter, phone contacted was attempted on three occasions. All procedures were conducted in accordance with the Declaration of Helsinki and approved by the Monash University Human Research Ethics Committee. Written informed consent was obtained from all participants.

### Statistical analysis

Data relating to the characteristics of participants, their level of pain and disability, and their beliefs about back pain were tabulated. Univariable and multivariable analyses for pain and disability were performed using ordinal regression [14], as the outcome variables pain and disability were categorised into ascending levels based on the CPG methodology. However, the assumption of proportional odds on which this method was based was violated for the pain outcome when two independent variables, menopause status and back beliefs, were included in the multivariable model. Thus, we applied the generalized partial proportional odds model described by Williams (2006)[15] to fit non-parallel regression lines for the three pain categories. A parallel regression line was used for other variables and hence they share the same regression coefficients. Given the proportional odds assumption was held for the disability outcome, only the parallel regression line was fitted. Multivariable analyses involved adjustment for the following potential confounders; age, BMI, smoking, alcohol consumption, and menopause, relationship and employment status. The univariable analysis was performed using the ordinary ordinal regression method [14]. The statistical analyses were conducted using Stata software version 9.2 [16]. A statistical test was considered to be significant if the associated p-value was less than 0.05.

## Results

Of the 542 subjects who agreed to take part in this study, 506 (93.4%) returned the study questionnaire. Their median (minimum, maximum) age and BMI was 58 (24, 80) years and 26.4 (15.6, 54.0) kg/m^2 ^respectively (Table 1). 91.9% of the women reported they have 'ever' experienced back pain, while 69.7% and 75.1% reported back pain in the past 6 and 12 months respectively, and 22.5% reported back pain 'now' (at the time of questionnaire completion). According to the CPG, 140 (27.7%), 284 (56.1%) and 82 (16.2%) women reported no, low and high pain intensity respectively, while 398 (78.7%), 71 (14.0%) and 37 (7.3%) women were classified as having no, low and high disability.

**Table 1:** Characteristics of study participants (n = 506)

**Participant characteristics**	**n (%)**
**Age **(years)^†^	58 (24, 80)
**Body mass index (BMI) **(kg/m^2^)^†€^	26.4 (15.6, 54.0)
**Smoking status: **Yes**°**	52 (10.3)
**Alcohol consumption: **Yes^±^	405 (80.7)
**Partner status**: Partnered^#^	342 (67.7)
**Employment status**: Employed^¥^	260 (53.3)
**Menopause status: **Postmenopausal	328 (64.8)
**Back pain prevalence:**	
Now	114 (22.5)
6 months^∝^	352 (69.7)
12 months	380 (75.1)
Ever	465 (91.9)
**Level of pain intensity:**	
No	140 (27.7)
Low	284 (56.1)
High	82 (16.2)
**Level of disability:**	
No	398 (78.7)
Low	71 (14.0)
High	37 (7.3)

^†^Median (minimum, maximum); ^€^BMI: 40 cases missing data; **°**Smoking status: 1 case missing data; ^± ^Alcohol consumption: 4 cases missing data; ^#^Partner status: 1 case missing data; 'Partnered' refers to participants that were married, in a de-facto relationship or single with a partner. ^¥ ^Employment status: 18 cases missing data. Employment excluded voluntary work or unpaid work in the home. ^∝ ^Past 6 months: 1 case missing data.

### Beliefs about back pain

The mean (SD) belief score for the cohort was 30.7 (6.0) (Table 2). Of the 9 statements examining beliefs about back pain, over 30% of women agreed or strongly agreed with 4 negative statements. Of note, 50% of women agreed that 'once you have had back trouble there is always a weakness' and at least 30% agreed that 'back trouble means periods of pain for the rest of one's life', 'back trouble makes everything in life worse' and 'later in life back trouble gets progressively worse'. A further 16% and 18% of women agreed that 'back trouble will eventually stop you from working' and 'back trouble must be rested' respectively.

**Table 2:** Percentage of participants that responded negatively to statements (i.e. selected agree or completely agree (4 or 5)) about back pain from the Back Beliefs Questionnaire (n = 506)

**Back Beliefs Questionnaire statements**		Participants with negative beliefs* n (%)
1	There is no real treatment for back trouble	49 (9.7)
2	Back trouble will eventually stop you from working	81 (16.0)
3	Back trouble means periods of pain for the rest of one's life	154 (30.4)
4	Back trouble makes everything in life worse	164 (32.4)
5	Back trouble may mean you end up in a wheelchair	58 (11.5)
6	Back trouble means long periods of time off from work	49 (9.7)
7	Once you have had back trouble there is always a weakness	252 (49.8)
8	Back trouble must be rested	91 (18.0)
9	Later in life back trouble gets progressively worse	180 (35.6)
	**Total score (mean, SD)**	30.7 (6.0)

* Selected agree or completely agree, which is a score of 4 or 5 respectively.

### Beliefs about back pain and self-reported pain intensity

The mean (SD) belief scores for women with no, low and high pain intensity were 30.2 (6.3), 31.5 (5.8) and 28.5 (5.7) respectively (Table 3). There were differences in the proportion of negative responses between individuals with no, low and high pain intensity for 5 of the 9 statements. However, while the high pain intensity group had the greatest proportion of negative responses for 3 of the statements, more women in the group with no pain responded negatively to 2 statements; 'back trouble makes everything in life worse' and 'back trouble means long periods of time off from work'.

**Table 3:** Association between beliefs about back pain and self-reported low back pain intensity

**Back Beliefs Questionnaire Statements**	Participants with negative beliefs about back pain* n (%)			**Univariable analysis** **(Chi-square)**
	**No pain** (n = 140)	**Low pain intensity** (n = 284)	**High pain intensity** (n = 82)	

There is no real treatment for back trouble	12 (8.6)	24 (8.5)	13 (15.9)	0.1
Back trouble will eventually stop you from working	22 (15.7)	36 (12.7)	23 (28.0)	0.004
Back trouble means periods of pain for the rest of one's life	28 (20.0)	84 (29.6)	42 (51.2)	<0.0001
Back trouble makes everything in life worse	56 (40.0)	80 (28.2)	28 (34.1)	0.047
Back trouble may mean you end up in a wheelchair	22 (15.7)	24 (8.5)	12 (14.6)	0.05
Back trouble means long periods of time off from work	23 (16.4)	18 (6.3)	8 (9.8)	0.004
Once you have had back trouble there is always a weakness	64 (45.7)	143 (50.4)	45 (54.9)	0.4
Back trouble must be rested	27 (19.3)	48 (16.9)	16 (19.5)	0.8
Later in life back trouble gets progressively worse	42 (30.0)	96 (33.8)	42 (51.2)	0.004
**Total score (mean, SD)**	30.2 (6.3)	31.5 (5.8)	28.5 (5.7)	0.051

* Selected agree or completely agree, which is a score of 4 or 5 respectively

### Beliefs about back pain and self-reported disability

The mean (SD) belief scores for women with no, low and high disability were 31.3 (5.7), 30.2 (6.3) and 24.8 (5.7) respectively (Table 4). Women with high level disability had more negative responses for 4 of the 9 belief statements than those with no or low disability. These included 'back trouble will eventually stop you from working' (p < 0.0001), 'back trouble means periods of pain for the rest of one's life' (p < 0.0001), 'back trouble means long periods of time off from work' (p = 0.02) and 'later in life back trouble gets progressively worse' (p = 0.002).

**Table 4:** Association between beliefs about back pain and self-reported low back disability

**Back Beliefs Questionnaire statements**	Participants with negative beliefs about back pain* n (%)			**Univariable analysis** **(Chi-square)**
	**No disability** (n = 398)	**Low disability** (n = 71)	**High disability** (n = 37)	

There is no real treatment for back trouble	33 (8.3)	9 (12.7)	7 (18.9)	0.07
Back trouble will eventually stop you from working	46 (11.6)	16 (22.5)	19 (51.4)	<0.0001
Back trouble means periods of pain for the rest of one's life	104 (26.1)	21 (29.6)	29 (78.4)	<0.0001
Back trouble makes everything in life worse	123 (30.9)	24 (33.8)	17 (45.9)	0.2
Back trouble may mean you end up in a wheelchair	42 (10.6)	8 (11.3)	8 (21.6)	0.1
Back trouble means long periods of time off from work	37 (9.3)	4 (5.6)	8 (21.6)	0.02
Once you have had back trouble there is always a weakness	194 (48.7)	34 (47.9)	24 (64.9)	0.2
Back trouble must be rested	68 (17.1)	12 (16.9)	11 (29.7)	0.2
Later in life back trouble gets progressively worse	128 (32.2)	30 (42.3)	22 (59.5)	0.002
**Total score (mean, SD)**	31.3 (5.7)	30.2 (6.3)	24.8 (5.7)	<0.0001

* Selected agree or completely agree, which is a score of 4 or 5 respectively.

### Associations between beliefs about back pain and pain intensity and disability

Multivariable analyses revealed a non-linear relationship between beliefs about back pain and self-reported pain intensity (Table 5). When comparing women with low and high pain intensity, there was an association, although not strong, between negative beliefs and high levels of pain, after adjusting for confounders (OR = 0.93, 95% CI: 0.88, 0.99; p = 0.02). However, there was no association between back pain beliefs and pain intensity when comparing those women with no and low intensity pain (OR = 1.03, 95% CI: 0.99, 1.07; p = 0.1). There was also a statistically significant, but not strong, association between beliefs about back pain and levels of self-reported disability, after adjusting for confounders, with more negative beliefs being associated with high levels of disability (OR = 0.93 (95% CI: 0.89, 0.97), p = 0.001) (Table 5).

**Table 5:** Associations between beliefs about back pain and self-reported levels of low back pain intensity and disability

**Participant characteristics**	**Pain intensity**				**Disability**			
	**Univariable analysis**		**Multivariable analysis^#^**		**Univariable analysis**		**Multivariable analysis**	

	Odds ratio (95% CI)	p value	Odds ratio (95% CI)	p value	Odds ratio (95% CI)	p value	Odds ratio (95% CI)	p value

Age (years)*	0.89 (0.78, 1.02)	0.1	0.67 (0.50, 0.89)	0.007	1.08 (0.91, 1.28)	0.4	0.94 (0.66, 1.34)	0.7
BMI (kg/m^2^)*	1.26 (1.07, 1.48)	0.005	1.22 (1.02, 1.45)	0.03	1.73 (1.43, 2.10)	<0.0001	1.61 (1.31, 1.98)	<0.0001
Smoking status	1.16 (0.67, 2.00)	0.6	0.99 (0.53, 1.87)	1.0	0.85 (0.42, 1.76)	0.7	0.99 (0.45, 2.16)	1.0
Alcohol intake	1.26 (0.81, 1.96)	0.3	1.75 (1.03, 2.97)	0.04	0.69 (0.42, 1.16)	0.2	1.10 (0.58, 2.12)	0.8
Partner status	1.28 (0.89, 1.84)	0.2	1.13 (0.74, 1.73)	0.6	1.02 (0.65, 1.61)	0.9	0.99 (0.57, 1.72)	1.0
Employment status	0.82 (0.58, 1.17)	0.3	0.62 (0.38, 1.01)	0.05	0.64 (0.41, 0.99)	0.045	0.74 (0.41, 1.35)	0.3
Menopause status^	0.91 (0.64, 1.30)	0.6	1.08 (0.53, 2.19)	0.8	1.19 (0.76, 1.85)	0.5	0.86 (0.36, 2.04)	0.7
Back pain now	20.8 (12.1, 35.7)	<0.0001	18.8 (10.3, 34.2)	<0.0001	4.78 (3.03, 7.55)	<0.0001	4.58 (2.71, 7.72)	<0.0001
Back beliefs score^	0.98 (0.96, 1.01)	0.3	1.03 (0.99, 1.07)	0.1	0.91 (0.88, 0.95)	<0.0001	0.93 (0.89, 0.97)	0.001
Menopause status	Low versus high pain intensity		3.24 (1.32, 7.96)	0.01				
Back beliefs score	Low versus high pain intensity		0.93 (0.88, 0.99)	0.02				

BMI – Body mass index. CI – confidence intervals. * Odds ratio per 10 year difference in age and 5 kg/m^2 ^difference in BMI. ^#^This multivariable analysis was performed using a partial proportional odds model [15], rather than ordinary ordinal regression [14]. ^ These variables demonstrated a non-parallel relationship with pain intensity, with data representing the comparison between no pain and low intensity pain groups

## Discussion

This study indicates that women recruited from a community setting generally have positive beliefs about back pain. However, within the study population, women who reported a high level of pain intensity and disability held more pessimistic views, which are not consistent with evidence-based practice.

The level of beliefs about back pain in our community dwelling women was similar to that reported in an Australian population-based study of people in 2000 [8] and 2002 [17]. However, our findings differ from more pessimistic beliefs reported by two population-based studies undertaken in Australia in 1997 [8] and Canada in 2005 [9]. These more negative results may be explained by the timing of these studies, as both were undertaken prior to the implementation of large public health campaigns about back pain. Taken together, these results indicate that our study population of women has a relatively optimistic view of back pain and its consequences.

We found negative beliefs were associated with high pain intensity in individuals living in the community. Previous studies have primarily focused on investigating the relationship between beliefs, measured by the Fear-Avoidance Beliefs Questionnaire, and pain intensity in low back pain patients receiving treatment. Although data examining the relationship between the Fear-Avoidance Beliefs Questionnaire and BBQ are limited, a cross-sectional study of 2,727 acute low back pain patients in primary care practice in France reported an association between pain intensity and fear-avoidance beliefs [18]. Moreover, a prospective cohort study of 123 patients with acute low back pain recruited from primary health care reported fear-avoidance beliefs to predict pain at 12 months, after adjusting for socio-demographic and pain variables [19]. While these data suggest that beliefs about back pain are predictive of long-term pain in a clinical population receiving treatment, it is unknown whether this is the case in the general population.

We found the relationship between beliefs about back pain and pain intensity to be dependent on the level of pain intensity. While there was no relationship between beliefs and pain intensity when comparing the no and low pain intensity groups, there was a clear association between these parameters for those with low and high pain intensity. Even though the difference between the mean beliefs scores for the low and high pain intensity groups was small, it is consistent with the degree of change in belief scores following the implementation of a successful public health intervention [17]. While it is possible that negative beliefs about back pain may lead to women reporting higher intensity pain, greater pain intensity may also result in the development of pessimistic beliefs about back pain. Our study provides evidence to indicate there is an association between back pain beliefs and pain intensity in the broader community and that further longitudinal investigation is needed to determine whether a causal relationship exists.

We found a clear association between back pain beliefs and levels of self-reported disability, with more negative beliefs being associated with high levels of disability. Although, to our knowledge, there are no population-based studies that have comprehensively examined the relationship between back pain beliefs and low back disability, our findings are consistent with studies of the general population that have examined other psychosocial factors, such as kinesiophobia and pain catastrophising [20,21] and clinical studies of patients with low back pain that have investigated fear-avoidance beliefs. For instance, a cross-sectional study of 443 patients with sub-acute low back reported an association between patients' fear-avoidance beliefs and perceived disability [22] and a prospective study of 83 patients with chronic low back pain reported fear-avoidance beliefs about physical activity to predict disability, after adjusting for confounders, such as pain intensity [23]. While these results indicate that negative beliefs may contribute to disability in acute low back pain, our understanding of whether beliefs lead to, or are a result of disability, in community-dwelling individuals is still unclear.

It is possible that our study may have been affected by selection bias. This may have occurred as we were limited to contacting women from our database who had agreed be involved in future research. However, we recruited subjects from a database established though random sampling from the state electoral roll, which includes all adults residing in the state, and without reference to research into back pain at the time of recruitment. In addition, the demographics of the women in the current study were very similar to those in the original study (mean (SD) age: 50 (14.4) years; mean (SD) BMI: 27.8 (6.5) kg/m2), even though the current study was conducted 4 years later. Although this cross-sectional study limits our ability to investigate factors such as pain duration and health care utilisation, explore predictors of low back pain, and generalise our results to the whole population, we have shown that both pain and disability are independently associated with beliefs.

## Conclusion

In conclusion, this study demonstrates that although community-dwelling women generally have positive beliefs about back pain, there are subgroups of women, with intense pain and disability, who hold pessimistic views about back pain which are not concordant with evidence-based practice. The association between back pain beliefs and both pain intensity and disability reported in this community-based study highlights the need for future longitudinal studies to determine the predictive nature of these relationships.

## Abbreviations

BBQ: Back Beliefs Questionnaire; BMI: body mass index; CPG: Chronic Pain Grade Questionnaire; SD: standard deviation; OR: odds ratio; CI: confidence intervals.

## Competing interests

The authors declare that they have no competing interests.

## Authors' contributions

DU: selected the questionnaires, co-ordinated the data collection, analysed the data and wrote the manuscript; RB: assisted with the co-ordination of the study, data analyses and interpretation and manuscript preparation; FC: assisted with data analyses and interpretation, and manuscript preparation; JC/AF: performed the statistical analysis and reviewed the manuscript. SD: provided access to an established research database, and assisted with co-ordination of the study, data interpretation and critical review of the manuscript.

## Pre-publication history

The pre-publication history for this paper can be accessed here:


